# Preparation of Polylactide/Halloysite-Nanoclay/Polytetrafluoro-Ethylene Composite Foam and Study of Properties and Morphology

**DOI:** 10.3390/nano15090667

**Published:** 2025-04-27

**Authors:** Silla George Raju, Hanieh Kargarzadeh, Andrzej Galeski

**Affiliations:** 1 BioMedChem Doctoral School, the University of Lodz and Lodz Institutes of the Polish Academy of Sciences, 21/23 Matejki Street, 90-237 Lodz, Poland; silla.george-raju@cbmm.lodz.pl; 2 Centre of Molecular and Macromolecular Studies, Polish Academy of Sciences, Sienkiewicza 112, 90-363 Lodz, Poland

**Keywords:** polylactide, nanocomposite, extrusion foaming, PTFE, nanoclay, strain hardening

## Abstract

Halloysite nanoclay (HNC) and as-polymerized polytetrafluoroethylene powder (PTFE) were introduced into biodegradable polylactic acid (PLA) via a melt mixing technique to enhance its mechanical, rheological properties and foaming ability. The synergetic effects of these fillers on the morphological, mechanical, thermal, and foaming properties of PLA were investigated. Results indicated that the tensile properties were improved in comparison to neat PLA. Differential Scanning Calorimetry (DSC) revealed a decrease in PLA crystallization time with increasing filler concentration, indicating a strong nucleating effect on PLA crystallization. Extensional flow tests showed that strain hardening in PLA composites is influenced by fillers, with PTFE particularly exhibiting a more pronounced effect, attributed to nanofibrillation and entanglement during melt processing. The addition of a dual-filler system improved the melt strength and viscosity of PLA, resulting in foams with decreased cell size and increased cell density.

## 1. Introduction

Polymer foams constitute an intriguing field of materials that offer numerous benefits, including lightweight properties, high strength, insulation, extensive surface area, damping, cost efficiency and many more [1,2,3]. Numerous petroleum based polymers, particularly polyurethane [4], polyethylene [5], polypropylene [6,7], and polystyrene [8], are utilized to produce foams. However, researchers have focused on minimizing pollution from these polymers by reducing the use of petroleum-based polymers and replacing them with environmentally acceptable biopolymers. Many biodegradable polymers, including poly(lactic acid), polyvinyl alcohol, polyhydroxyalkanoates and poly(butylene succinate), are being extensively studied. Among these, poly(lactic acid) (PLA) has acquired comparable mechanical properties to petroleum-based polymers, making it an alternative for various applications in diverse fields such as sensors [9], packaging [10], and biomedicine [11]. PLA is a semi-crystalline polymer characterized by exceptional qualities, including favorable biocompatibility, significant biodegradability, robust tensile strength, high availability, low toxicity, and enhanced thermal properties [12,13]. Nevertheless, commercially available PLA exhibits several drawbacks, especially inadequate melt viscosity, brittleness, elevated cost, low thermal resistance, and absence of melt strain hardening, rendering PLA unsuitable for the effective foam production.

Various strategies have been established to mitigate these disadvantages and enhance their features and applications. Techniques such as the inclusion of chain extenders, crosslinking, blending, grafting, and the incorporation of nucleating agents can enhance attributes like melt strength, cell density, cell structure, and melt strain [14,15]. The process of polymer blending is an efficient technique for enhancing rheological and foaming properties. Physical blending can enhance the toughness and thermal resistance of PLA [16,17]. Here, PTFE is chosen to improve the characteristic features of PLA. The shear force during blending converts PTFE into nanofibrils, forming an entangled network structure inside the matrix, which subsequently improves the crystallization and mechanical strength of the polymer. In situ fibrillation of PTFE as polymerized-flakes was first discovered in our lab [18,19,20,21,22,23]. Later, the in situ PTFE fibrillation was used by others as a routine way of improving melt strength of various polymer systems and in foaming applications, e.g., [24,25,26,27,28,29,30].

The in situ fibrillation of as-polymerized PTFE is possible because of low chain entanglement level and the ease of large deformation without strain hardening. The low level of chain entanglements is the effect of polymerization condition: PTFE is usually polymerized well below its crystallization temperature (200–240 °C vs. a crystallization temperature 310–320 °C). Then, as-polymerized fragments of a chain continue the attachment to the growing crystal rather than entangling with surroundings. However, any melting of PTFE crystals causes quick chain entanglements due to thermal motion of chain ends and chain fragments. Such recrystallized PTFE is unable to be in situ fibrillated due to strain hardening. Similar in situ fibrillation was achieved for polypropylene [31] and polylactide [32] when the entanglement level was decreased by precipitation from dilute solution and using polyethylene [33] that was polymerized, as it was for PTFE, at the low temperature of 10 °C, which is below its usual crystallization temperature of 90–110 °C. All those nanocomposites containing in situ-generated nanofibrils exhibit strain hardening of extensional viscosity. Hence, a disentangled polymer will form nanofibrils during blending and may serve as an adjuvant in foaming.

Here we performed in situ fibrillation of PTFE to increase PLA melt strength and to improve foaming process. Moreover, PTFE is a bio-inert substance, and the incorporation of a minimal quantity of PTFE into PLA would not influence their biological properties. PLA/PTFE foam with tunable pore morphology, enhanced storage modulus, superior crystallinity, and high compression strength which has a suitable application in cushioning materials was developed and reported recently by others [34,35].

The incorporation of clay nanoparticles can additionally augment the melt strength of PLA. Reports indicate that a homogeneous and fine cellular structure can be achieved by the appropriate clay composition [36]. Likewise, the uniform dispersion of nanoclay can yield foams with a closed cell structure, resulting in enhanced mechanical and thermal stability [37]. The literature indicates that nanoclay has been utilized as a filler in PLA to produce a uniformly denser composite with improved crystallinity [38]. Numerous studies have reported on the effect of a single filler in PLA. Nevertheless, limited studies have been conducted on PLA-based ternary nanocomposites. These investigations are crucial for comprehending how enhanced properties arise from the interplay of dual filler species. As of now, there have been no reports on the integration of PTFE/HNC into a PLA matrix. This work involved the melt mixing of PTFE and HNC with PLA at varying filler loadings to produce both composites and their foams using an extruder. This study aims to further understand the synergistic effects of PTFE/HNC filler in PLA on its morphological, mechanical, thermal, and foaming properties.

## 2. Materials and Methods

### 2.1. Materials

Poly(lactic acid) (PLA) pellets (IngeoTM 4032D) with a specific gravity of 1.24 g/cm^3^ and a melt flow index (MFI) of 6 g/10 min (210 °C, 2.16 kg) were purchased from NatureWorks LLC, Blair, NE, USA. Halloysite nanoclay (HNC) nanopowder with a density of 2.53 g/cm^3^ and a pore volume in the range of 1.26 to 1.34 mL/g was obtained from Sigma Aldrich. Poly(tetrafluoroethylene) (PTFE) powder, characterized by an average particle size of 28 μm and a melting point of 345.7 °C, was purchased from DuPont. The exothermic chemical blowing agent azodicarbonamide (ADC) (Porofor ADC/L-C2), which has a decomposition temperature of 214 °C and a volumetric gas production of 228 mL/g at 210 °C, was supplied by Lanxess (Cologne, Germany).

### 2.2. Preparation of Composites and Foams

All materials were oven-dried at 60 °C for 24 h prior to processing to eliminate the presence of any excess moisture. Neat PLA binary and ternary samples were melt-compounded with varying compositions of HNC and PTFE (as detailed in Table 1) using a co-rotating twin-screw extruder (Zamak Mercator, Skawina, Poland) at a fixed screw speed of 60 rpm for all samples. The temperature profile was set at 180, 185, 190, 195, 200, 200, 210, 210, 210, 210 °C from the hopper to the die. The produced extruded strands were 3–4 mm in diameter and were pelletized using a pelletizer. Followed by hot pressing at 190 °C for five minutes at a pressure of 20 MPa, 1 mm thick sheets were formed and then cooled to ambient temperature.

PLA foams were produced via extrusion foaming with the addition of 3 phr ADC in a co-rotating twin-screw extruder under similar aforementioned processing conditions. Additionally, neat PLA was also prepared with analogous processes to obtain a reference sample. All samples were stored overnight to ensure stability prior to characterization.

The designation of the samples is based on the proportion of HNC and PTFE present in the PLA matrix. For instance, PLA-H(0.5)P(1) denotes a PLA composite containing 0.5 wt% of HNC and 1 wt% of PTFE.

## 3. Characterization

### 3.1. Scanning Electron Microscopy (SEM)

The surface morphology of the materials was examined using a scanning electron microscope (SEM), JEOL JSM-5500 LV, operated in high vacuum mode at a 15 kV accelerating voltage. The fractured surface of the composites obtained after the tensile tests was utilized for analysis. For foam samples, the internal structure was exposed by breaking in liquid nitrogen. The exposed surface of the sample was coated with a 10 nm fine gold layer using ion sputtering (JEOL JFC-1200) prior to SEM examination.

### 3.2. Tensile Measurements

Tensile tests of the neat PLA and composites were performed using an Instron 5582 universal testing machine according to ISO 527-2. Dog-bone-shaped specimens with a gauge length of 25 mm and 5 mm width were cut out from compression-molded sheets using a steel template. All tests were conducted at room temperature with a load cell of 2 kN and a cross-head speed of 5 mm/min. The tensile measurements were obtained by averaging four runs for each composition.

### 3.3. Differential Scanning Calorimetry (DSC)

The thermal properties of the composites were studied using the Differential Scanning Calorimeter model Q20 from TA Instruments. Approximately 6 to 7 mg of each sample were encapsulated in an aluminum pan. An empty aluminum pan similar to the sample pan was used as the reference. The measurement followed a heat-cool-heat pattern. For non-isothermal crystallization, all samples were first subjected to heating from 40 °C to 200 °C, subsequently cooled to 40 °C, and then reheated at the same rate for the second heating scan. After the initial heating and cooling, the isothermal condition was maintained for 2 min at 200 °C and 40 °C, respectively. The whole heating process was carried out at a scan rate of 10 °C /min under a 50 mL/min nitrogen flow. The first heating scan was conducted to eliminate any previous thermal history. The thermal variables, including glass transition temperature (*T*_g_), crystallization temperature (*T*_c_), melting temperature (*T*_m_), crystallization enthalpy (Δ*H*_c_), and melting enthalpy (Δ*H*_m_), were calculated from the first thermal scan. The crystallinity ($X_{c}$) of PLA and the composites was obtained using the Equation (1):

(1) $$X_{c}\left(\%\right)=\frac{\Delta H_{c}}{\Delta H_{m}^{0}\times (1-W_{t})}$$

where **Δ*H*_c_** refers to the crystallization enthalpy of PLA/HNC/PTFE composites, $\Delta H_{m}^{0}$ is the enthalpy of melting of 100% crystals of PLA (93.6 J/g) [39], and $W_{t}$ is the filler weight fraction in PLA/HNC/PTFE composites.

For isothermal crystallization, the samples were first heated to 200 °C at a rate of 20 °C/min and then equilibrated for 3 min. Subsequently, the samples were cooled to a range of isothermal crystallization temperatures (80, 90, 100, 110, 120, 130, and 140 °C) at a rate of 20 °C/min and equilibrated until complete crystallization. Finally, the melting behaviour was analyzed by heating up to 200 °C at 20 °C/min.

### 3.4. Rheology Measurements

The rheological behaviour of the unfoamed samples was evaluated using a strain-controlled rotational rheometer (ARES LS2, TA Instruments, New Castle, DE, USA). The uniaxial extension tests of the molten samples were conducted using an extensional viscosity fixture (EVF, TA Instruments) attached to the ARES rheometer. Prior to the commencement of measurements, the samples were compression molded into rectangular strips measuring 18 × 10 × 0.7 mm^3^ at 170 °C in the standard mold, provided by TA Instruments with the EVF. The molten samples were subjected to uniaxial extension at a constant Hencky strain rate, $\dot{\varepsilon }$, of 0.1 s^−1^ at 175 °C.

### 3.5. Foam Cell Analysis

The density (ρ) of the ternary system, including both composite and foamed samples, was determined using Equation (2):

(2) $$\rho =\frac{w}{v}$$

where w represents the mass and v denotes the volume of each sample. The measurements were carried out using a pycnometer for measuring the volume of the samples. The experiment was performed three times to ascertain the average density.

Image analysis software (ImageJ ver. 1.53t) was employed to analyze the quantitative measurements such as cell density and cell size. The cell density ($N_{0})$ is calculated by Equation (3):

(3) N0=nA32ρcρf

where n represents the number of cells in the SEM image, and A is the area of the image (in cm^2^). $\rho_{c}$ and $\rho_{f}$ denote the densities of composite and foamed samples, respectively [40]. SEM images with ×75 magnification possessing approximately 120 to 170 cells per image were used for analysis. $V_{f}$ signifies the void fraction and was determined using Equation (4):

(4) Vf=1−ρfρc

## 4. Results and Discussions

**Figure 1:**
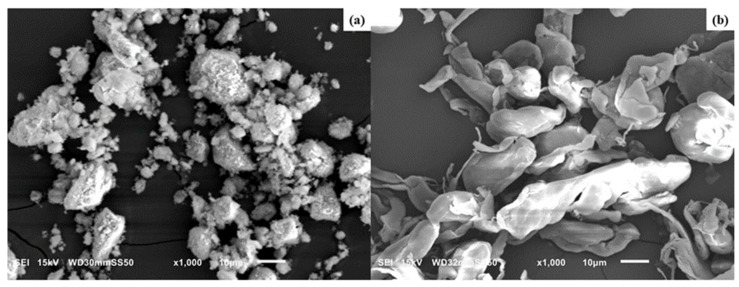
SEM images of (**a**) HNC and (**b**) PTFE particles.

SEM images of HNC and PTFE particles are shown in Figure 1a,b, respectively.

The HNCs are found to be aggregated in their natural state, possessing a high specific area and strong van der Waals force among each HNC particle. This aggregation can affect the bonding between the filler and the polymer matrix, which can adversely affect the mechanical property of the composite. An agglomeration of halloysite nanoparticles can be reduced either by soft acid etching or by organic modification (see for example [41]). The PTFE particles before PLA melt-mixing (Figure 1b) were flakes or irregular-shaped grains with varying size and are individually separated. According to the manufacturer, the as-polymerized grains were mechanically rolled before supplying the material under the name of Teflon 7C.

### 4.1. Fracture Surface Morphology of PLA Composites

**Figure 2:**
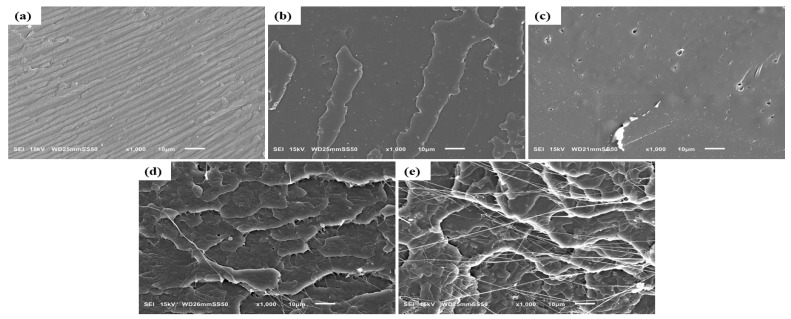
SEM image of the fractured surface of (**a**) Neat PLA and binary composites: (**b**) PLA-H(0.5), (**c**) PLA-H(3), (**d**) PLA-P(0.5), and (**e**) PLA-P(3).

**Figure 3:**
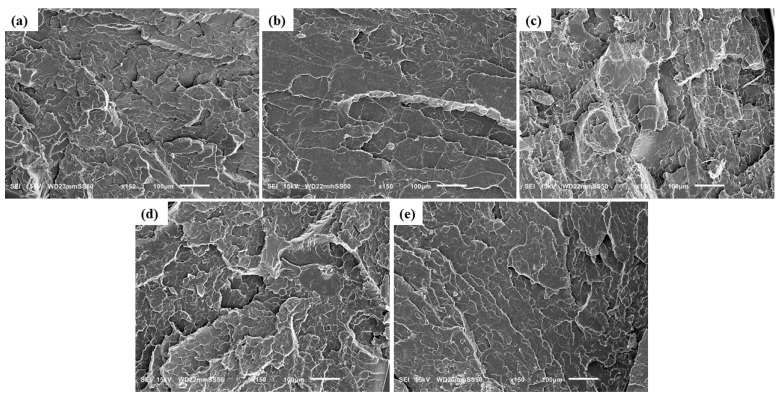
SEM image of the fractured surfaces of ternary composites: (**a**) PLA-H(0.5)P(0.5), (**b**) PLA-H(0.5)P(1), (**c**) PLA-H(0.5)P(3), (**d**) PLA-H(1)P(0.5), (**e**) PLA-H(3)P(0.5).

The fracture surfaces of neat PLA and its binary and ternary melt compounded composites with HNC and PTFE were studied by SEM, as illustrated in Figure 2 and Figure 3. Fracture resulted from the tensile tests. The neat PLA (Figure 2a) displays a grooved surface which corresponds to its brittle facture and crazing behavior. A uniform dispersion of HNC was observed on the surface of PLA composites with 0.5 wt% (Figure 2b). For higher concentrations of HNC it can be seen that the surface in Figure 2c possess tiny porous inner structures on the fractured surface, which is due to the aggregation of HNC particles, causing a negative effect on their mechanical property enhancement. The significant rough surfaces in Figure 2d,e result from the incorporation of PTFE into the system, leading to their plastic deformation during PLA melt compounding.

However, it is seen that the PTFE particles become fibrillated (Figure 2e) after they are processed, similarly reported in the literature [18,19,20,21,22,23,42].

For ternary composites, a relatively uniform dispersion of HNC and PTFE is observed in the PLA-H(0.5)P(0.5) composite (Figure 3a). The presence of a small number of fibrils in the composite is attributed to the lower filler concentration in the matrix. Increasing the PTFE concentration to 1 wt% results in the generation of more fibrils (Figure 3b). During melt mixing, the substantial shearing force facilitates the formation of PTFE nanofibrils by overcoming the weak cohesive forces inherent in the pseudo-hexagonal polymorphic crystal structure of PTFE, thus promoting fibrillation [20]. Consequently, the compounding of the PLA melt with PTFE led to a nano-fibrillar entangled network structure being formed [19,21,27]. Furthermore, this enhances the foamability of the composite due to the strong rheological reinforcement provided by PTFE nanofibrils [21,23].

A relatively homogeneous distribution of HNC was observed on the surface of ternary composites at concentrations of up to 1 wt% (Figure 3d). This demonstrates the ability of halloysite nanoclay to effectively disperse at low concentrations. An optimal distribution of halloysite nanoclay inside the polymer matrix enhances the mechanical characteristics of the material. Nevertheless, when the concentration exceeds 1 wt%, aggregation of halloysite nanoclay occurs (Figure 3e), adversely affecting the enhancement of mechanical properties. The extensive surface area and robust van der Waals forces lead to the aggregation of nanoscale fillers. Consequently, concentration is a critical factor influencing the distribution of filler inside a matrix. Higher concentration leads to agglomeration, while low concentration can provide effective dispersion. Analogous outcomes of filler aggregation with the use of high concentrations of nanoparticles in the PP/NC/NCC ternary system have been previously reported in the literature [43]. As the filler content of PTFE reaches 3 wt% (Figure 3c), prominent aggregates of HNC and thicker PTFE fibrils are found. Nevertheless, apart from PLA-H(0.5)P(3) and PLA-H(3)P(0.5), no other ternary composite developed nanoparticle agglomeration, indicating that HNC and PTFE fillers are effectively dispersed throughout the PLA matrix during melt compounding. Moreover, PTFE fibrillation increases the material viscosity, resulting in the enhanced dispersion of HNC throughout the PLA/HNC/PTFE composite. The increase in viscosity results from the elevation of shear force, facilitating the homogeneous dispersion of nanoparticles during melt compounding. Huang et al. similarly reported the dispersion of CNT with the formation of fibrillated PTFE in a PBS/CNT/PTFE ternary composite [28]. In our older research we reported the formation of thinner PTFE nanofibers for shearing with a higher rate and for prolonged melt mixing time in the compounding of PTFE with polypropylene [20].

### 4.2. Tensile Properties

**Figure 4:**
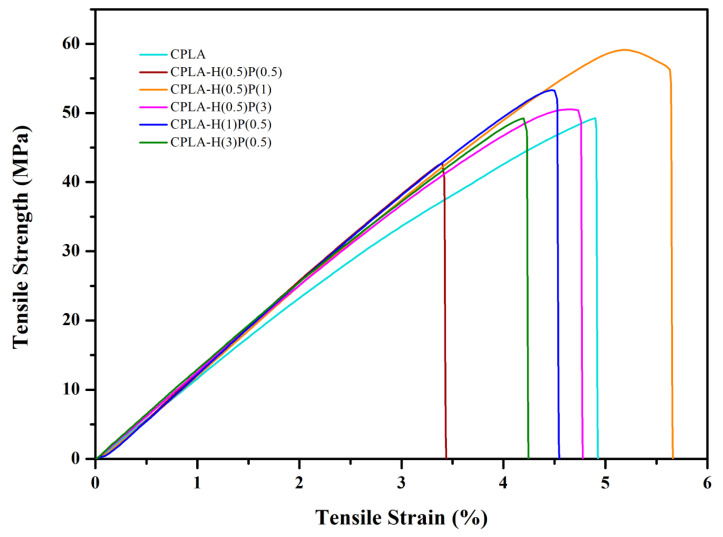
Stress–strain curves of neat PLA and PLA/HNC/PTFE ternary composites.

In order to analyze the mechanical properties of the prepared composites, tensile tests were performed and the obtained data are given in Table 2. Different ratios of HNC and PTFE were added to enhance the mechanical properties of neat PLA. Figure 4 demonstrates the stress–strain curve of neat PLA and PLA/HNC/PTFE ternary composites.

The incorporation of HNC and PTFE fillers increased the values of tensile strength and tensile strain for PLA-H(0.5)P(1). Quantitively, the tensile strain increased by approx. 15% from 4.9% to 5.7%, tensile strength increased by 20% from 49.3 MPa to 59.1 MPa, and Young’s modulus for PLA-H(3)P(0.5) increased from 1140 MPa to 1360 MPa, compared to neat PLA composite. The simultaneous increase of these parameters is due to the synergistic reinforcement effect of HNC and PTFE nanofibrils on the PLA matrix. The incorporation of an optimal amount of filler can significantly influence interfacial interaction between nanofiller and a polymer matrix, as observed for PLA/halloysite system [44].

The stiff nature of HNC can lead to the enhancement of the mechanical strength of the composite. Guo et al. reported the effect of HNT in improving the mechanical strength of PLA matrix at low loadings [45]. Also, the formation of fibrillated PTFE in the PLA matrix can cause plastic deformation in the PTFE’s vicinity, thereby creating stronger composites [46]. PLA-H(0.5)P(1) showed the highest tensile strength of 59.1 MPa. However, the tensile strength gradually decreased for all other filler ratios. This reveals that PLA-H(0.5)P(1) is the optimum concentration for the PLA/HNC/PTFE system which is attributed to the uniform dispersion and most favorable interfacial compatibility between halloysite nanoclay, PTFE, and PLA. The enhancement in tensile strength results is due to the increase in contact surface area and the interaction between the filler and the polymer matrix. This result is also in support of the SEM morphology. The addition of a higher concentration of filler can lead to agglomerates which form premature cracks due to the excessive stress concentration around agglomerates and poor adhesion between nanoclay, PTFE, and PLA. This consequently reduces the tensile strength of the composites.

**Figure 5:**
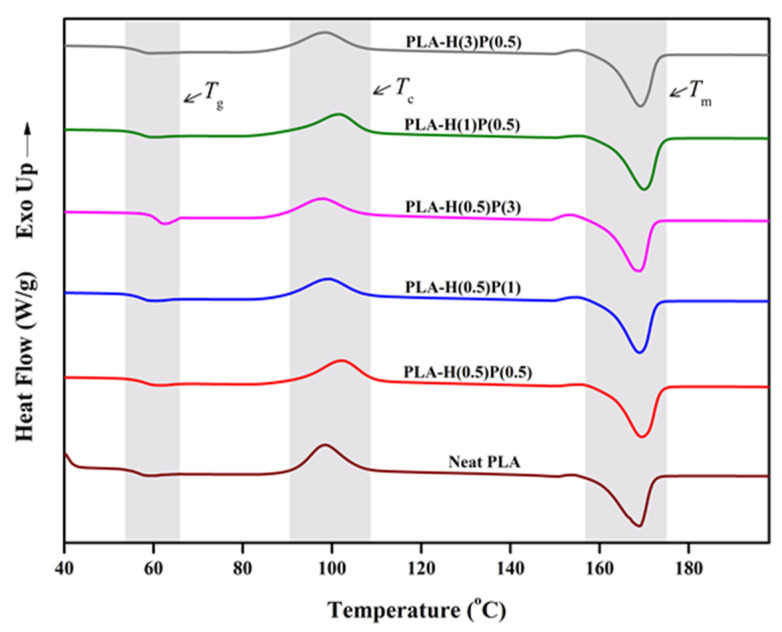
DSC thermogram of neat PLA and PLA/HNC/PTFE ternary composite of first heating scan.

**Figure 6:**
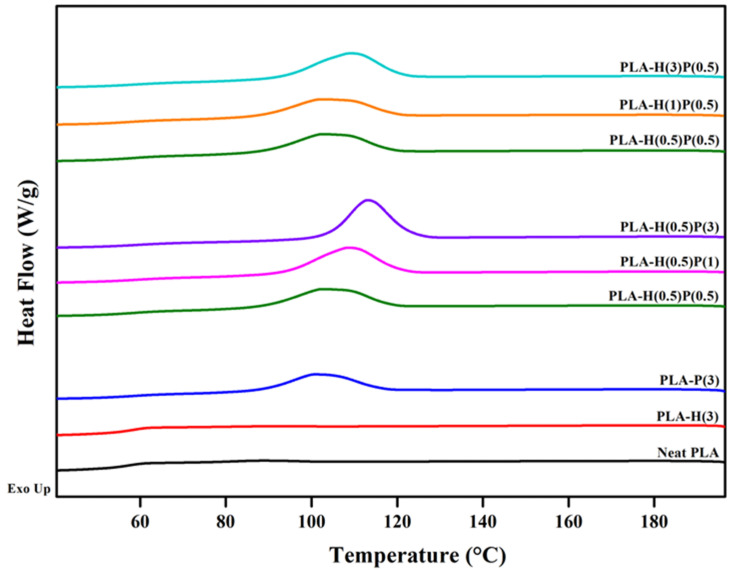
Crystallization exotherms for PLA and PLA nanocomposites during cooling from 200 °C at the rate of 10 °C/min.

**Figure 7:**
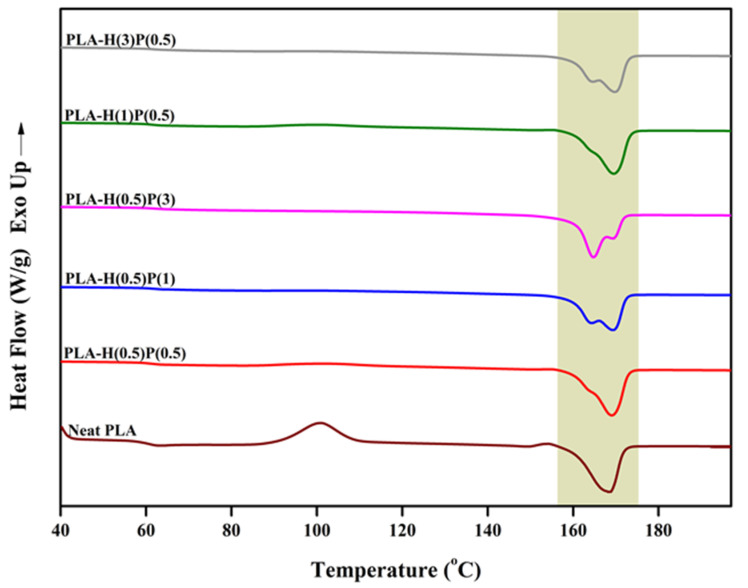
DSC thermogram of neat PLA and PLA/HNC/PTFE ternary composites’ second heating scan.

Young’s modulus asserts the stiffness of the material subjected to small deformation and signifies the capability of the material to respond to deformation. However, the addition of filler in PLA dropped Young’s modulus of ternary composite, leading to a lower stiffening effect and decreased structural rigidity in PLA molecular chains. This is in agreement with a low degree of crystallinity as determined by the DSC analysis. Please refer to Section 4.3 where the so-called “cold-crystallization” is observed on DSC heating curves in Figure 5, Figure 6 and Figure 7. This is an indication of incomplete crystallization of PLA and the ternary nanocomposites. A similar trend of decrease in the modulus was reported by Kamaludin et al. in chitosan/halloysite nanotube composites in PLA [47].

In the case of PLA/HNC/PTFE, the addition of fillers caused a decline in elongation at break for the ternary samples. This can be due to the stiffening effect of the filler and particle aggregation of HNC and PTFE which hinders, in any case at a low room temperature, the ductility of the molecular chains of PLA. Some limited aggregation of HNPs is distinctly seen in Figure 3c as white spots on an otherwise smooth surface exposed by brittle fracture in tensile test. The addition of a filler causes strong interaction with the polymer matrix, resulting in stretching resistance upon strain. However, the addition of 1 wt% of PTFE into PLA/HNC increased the elongation at break, which may be the result of good dispersion of HNC and PTFE, along with the good interfacial bonding between PTFE and PLA (see SEM images in Figure 2d,e).

### 4.3. Thermal Properties

DSC analyses were performed to further investigate the impact of HNC and PTFE on the thermal characteristics of PLA, with the resultant thermograms during heating scans depicted in Figure 5 and Figure 6. The glass transition temperature (*T*_g_), followed by cold crystallization temperature (*T*_c_) and melting temperature (*T*_m_), are observed in the DSC thermogram. These thermal patterns are commonly exhibited by most of the semi-crystalline polymers, including PLA [48].

Four phenomena are observed on the thermogram presented in Figure 5: glass transition, *T_g_*, at a temperature around 60 °C, enthalpy relaxation just above *T_g_*, the cold crystallization of PLA with a peak at around 100 °C, and its melting at around 170 °C. The relaxation of enthalpy [49] corresponds to its release upon heating at and above *T_g_* after the material has been aged below its glass transition temperature [50]. The amplitude of the relaxation enthalpy depends on aging temperature and time, i.e., it depends on a sample history kept below the glass transition temperature. The crystallinity of those samples was settled during poorly controlled cooling after compounding. Generally, not all those specimens were fully crystallized, as is evident from the “cold crystallization” peak at around 90–110 °C. For all samples tested there are small shoulders at 150 °C and above but below the main melting peak at around 170 °C. That shoulder is attributed to the recrystallization of α’-crystals to α-crystals. Then, during further heating, all samples undergo melting. The melting concerns all crystals, those formed during compounding and cooling as well as those crystalized during cold crystallization. The detailed general knowledge on crystallization and melting behavior of PLA is extensively described by Tashiro and Tsuji [51].

After the first heating course the samples were cooled down in a controlled way with 10 deg/min and they underwent crystallization. The crystallization plots for the samples tested are presented in Figure 6.

The scans are collected in three groups: the first contains scans for plain PLA, PLA compounded with halloysite, and PLA compounded with PTFE. Cooling at the rate of 10 °C/min is too fast to trigger the strong crystallization of plain PLA and PLA compounded with halloysite. However, through comparison of the three curves it is clear that PTFE nucleates the crystallization of PLA, while halloysite is nearly neutral in terms of the crystallization of PLA. The exotherms of crystallization in the second group are for nanocomposites containing 0.5% of halloysite with an increasing content of PTFE. It is clear that by increasing the content of PTFE the onset of crystallization is shifted to a higher temperature, and also the intensity of crystallization is increased. The third group of exotherms is for the fixed concentration of PTFE, 0.5%, and the increasing concentration of halloysite. The three curves are of similar shape and similar height, but only for the highest concentration of halloysite is the peak slightly shifted to a higher temperature.

The second heating runs, presented in Figure 7, for the same set of samples were related to the samples with an identical thermal history.

The *T*_g_ of PLA and all nanocomposites were around 58–59 °C without a trace of enthalpy relaxation. A significant cold crystallization peak is observed for plain PLA, and small traces of cold crystallization is seen on DSC curves for composites with a low content of PTFE: samples H(1)P(0.5), H(0.5)P(0.5), and very tiny cold crystallization for H(3)P(0.5). The samples containing a higher concentration of PTFE (H(0.5)P(1) and H(0.5)P(3)) do not exhibit a cold crystallization peak during the second melting run. Further heating reveals some slight α’ PLA crystals reorganization to α crystals for the plain PLA only (see Ref. [51] for details of the mechanisms). All other samples do not show α’ presence and its reorganization. The melting of the samples in the range of 160 to 173 °C involves rearrangement, recrystallization, and finally the melting of α crystals. Such processes result in two endothermic peaks, as seen in Figure 6. The total enthalpy of melting was between 37 and 41 J/g for all samples tested, with slightly higher values for samples containing less PTFE and lower for samples containing a higher amount of PTFE.

**Figure 8:**
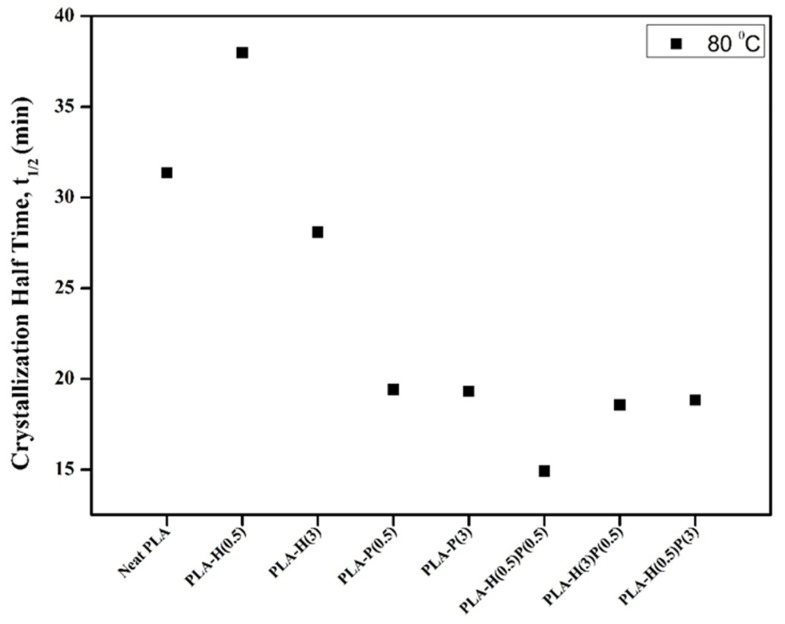
Half time crystallization (t_1/2_) at 80 °C of neat PLA, binary, and ternary composites.

The half-time crystallization is usually measured because it includes both the changes in the nucleation of crystallization and the changes in the onset of crystallization. In Figure 8 the half-time crystallization data of PLA and all composites are shown. The samples were melted by heating to 200 °C and then cooling down to 80 °C to register the crystallization heat release.

It is seen from the graph in Figure 8 that the addition of even a small amount of PTFE causes a decrease of half-time crystallization of the main components of PLA, while the incorporation of halloysite is rather inert for the crystallization of PLA. However, the addition of halloysite along with the addition of PTFE may lead to a better fibrillation and dispersion of PTFE, as it can be concluded from the lower half-crystallization time (15 min) for nanocomposite containing 0.5% of PTFE supplemented with 0.5% of halloysite compared to nanocomposite containing only 0.5% of PTFE (19 min).

Compared to neat PLA, a variation in crystallization and melting temperatures is seen, indicating that the thermal properties are affected by differing filler contents. The crystallization peak is observed to be displaced towards higher temperatures, indicating that the addition of fillers significantly influenced the crystallization behavior. This may be attributed to the nucleation ability of PTFE, and when combined with HNC, to function as nucleating agents. Hence, PTFE nanofibrils can help to promote the crystallization of PLA. As a result, the addition of PTFE combined with halloysite will shorten the solidification time of PLA nanocomposites during cooling and foaming. This can significantly change the foaming behavior of PLA nanocomposites.

### 4.4. Rheological Properties

**Figure 9:**
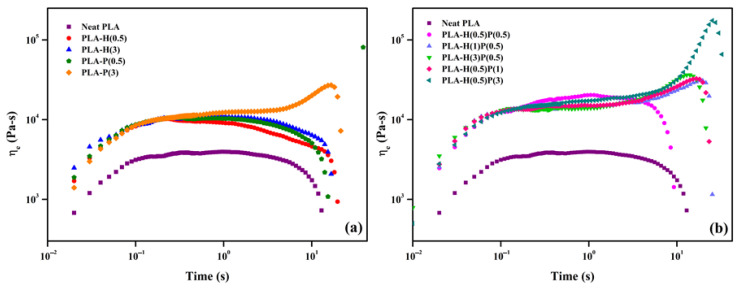
Extensional viscosity vs. time for (**a**) binary and (**b**) ternary composites.

The extensional flow tests were performed to analyze the rheological behavior of the PLA system influenced by HNC and PTFE. Figure 9 illustrates the variation in extensional viscosity as a function of time for neat PLA and their binary and ternary composites. Strain hardening was not observed for neat PLA and binary samples except 3 wt% of PTFE, which showed significant strain hardening behavior. Similar, results were found for ternary composites, where the strain hardening was found in higher concentrations of PTFE than compared to the HNC present. This signifies that the presence of PTFE was much more effective than that of HNC for the strain hardening behavior. This is because the PTFE nanofibrillar network undergoes deformation by the extensional flow, however, extension is further resisted by the fibrillar entanglements which produce a strain hardening effect [21,24].

Strain hardening is important for foaming as the bubble walls undergo extension during the bubble growth. Strain hardening prevents excessive growth and allows faster bubble fixation and foam solidification. Evidently, the tertiary nanocomposites: PLA-H(0.5)-P(3), PLA-H(3)-P(0.5), and PLA-H(0.5)-P(1) exhibit the highest strain hardening and should be best the composites for foaming.

### 4.5. Cellular Morphology of PLA Ternary Foams

**Figure 10:**
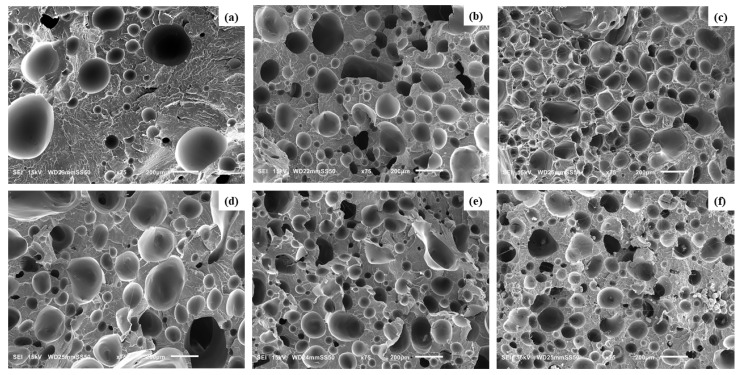
SEM images of foamed PLA samples (**a**) Neat PLA, (**b**) PLA-H(0.5)P(0.5), (**c**) PLA-H(1)P(0.5), (**d**) PLA-H(3)P(0.5), (**e**) PLA-H(0.5)P(1), and (**f**) PLA-H(0.5)P(3).

The cellular morphology of foamed PLA and PLA/HNC/PTFE, illustrating the effects of HNC and PTFE nanofibrils within the PLA matrix, is presented in Figure 10. The ternary foams demonstrate improved cell morphologies compared to pure PLA foam, as expected. This is because the fillers have potential reinforcement and nucleating effects, thereby complementing cell nucleation and cell proliferation. According to classical nucleation theory, the inclusion of nucleating agents creates heterogeneous nucleation sites by reducing the surface energy of polymer–particle interface [52,53]. This facilitates the production of a greater number of nuclei at reduced free energies [54,55].

Furthermore, HNC and PTFE acting as nucleating agents for PLA/HNC and PLA/PTFE nanocomposite foams, respectively, have been previously reported in the literature [26,56]. Consequently, a notable reduction in average cell size and an increase in cell density relative to the pure PLA foam are seen. In addition to this, the development of more refined cell morphology in ternary foams may be attributed to additional factors. Melt strength and melt viscosity of the polymer matrix are two critical parameters influencing cell growth [57]. The incorporation of HNC enhances the stiffness of the molecular chain and hinders the mobility of PLA chains. The formation of the fibrillated network by PTFE during melt blending also improves the melt viscoelasticity of the matrix. This slows bubble growth and inhibits cell coalescence and cell collapse. Ensuring appropriate polymer melt strength before foaming leads to consistent cell nucleation and the stable proliferation of cells with reduced cell size [58,59,60]. A comparable rationale has been documented in the research conducted by Zhao et al. on PP/talc/PTFE microcellular injected foams [61]. The SEM micrographs of sections across the foams obtained from nanocomposites are depicted in Figure 10.

**Figure 11:**
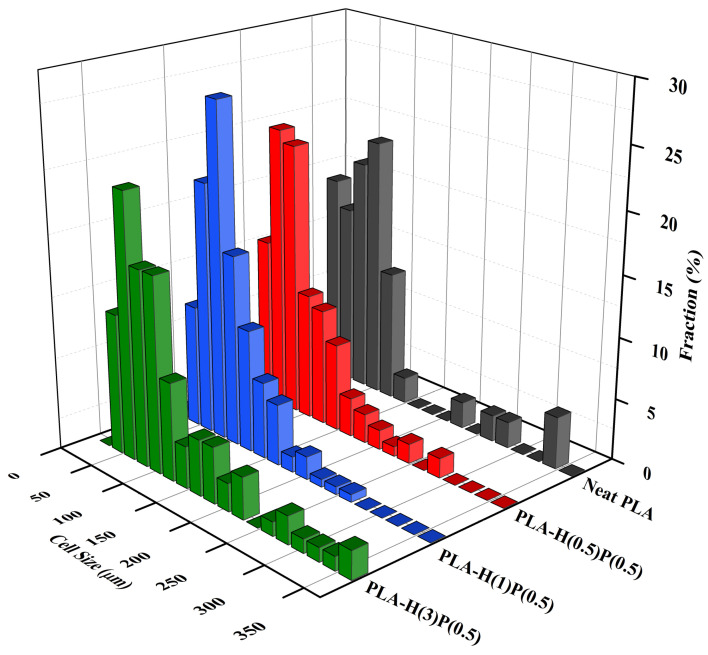
Cell size concentration graphs of foamed PLA nanocomposites consisting of a fixed amount of PTFE (0.5%) and increasing amount of halloysite: PLA-H(0.5)P(0.5), PLA-H(1)P(0.5), and PLA-H(3)P(0.5). A foam of neat PLA is included for comparison.

**Figure 12:**
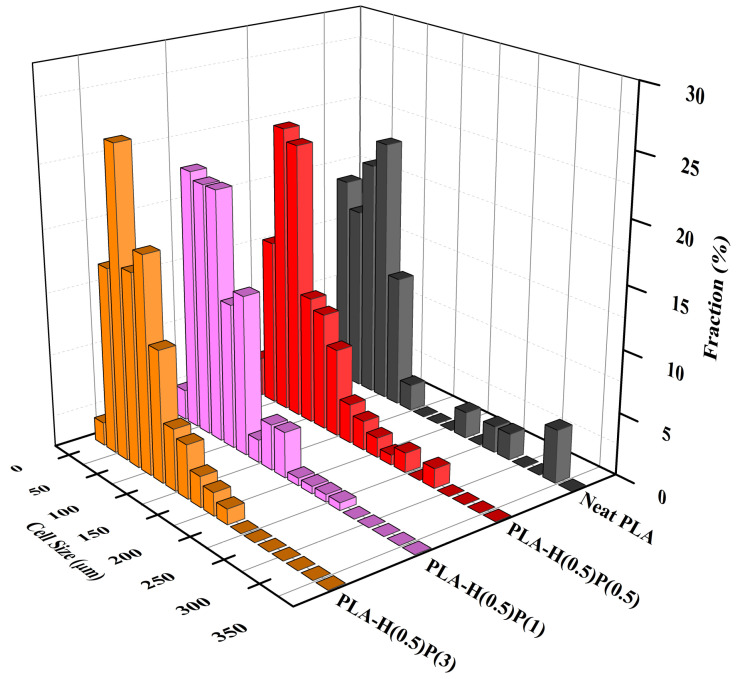
Cell size concentration graphs of foamed PLA nanocomposites consisting of a fixed amount of halloysite (0.5%) and increasing amount of PTFE: PLA-H(0.5)P(0.5), PLA-H(0.5)P(1) and PLA-H(0.5)P(3). A foam of neat PLA is included for comparison.

The sizes of bubbles seen in SEM micrographs in Figure 10 were measured and analyzed, leading to the number size distributions presented in Figure 11 and Figure 12.

In Figure 11, the cell size distributions for an increasing amount of halloysite are presented. By adding 0.5% halloysite the number of large bubbles is reduced as compared to neat PLA, which can be attributed to the nucleation of new bubbles by halloysite particles. Further increasing the amount of halloysite to 1% reduces even more the presence of large bubbles, which is a reaffirmation of the nucleation of new bubbles by halloysite particles. However, the further increase of halloysite concentration to 3% causes the reappearance of large and very large bubbles, which is attributed to the agglomeration of halloysite particles and breaking the bubble walls. In other words, agglomerates of halloysite particles act as an antifoaming agent if not sufficiently counteracted (by PTFE nanofibrils). Breaking the walls, halloysite is an antifoaming agent for larger concentration.

The foams that are analyzed in Figure 12 contain 0.5% of halloysite, which was sufficient for nucleate new bubbles but sufficiently low to form agglomerates that would act as an antifoaming agent. By increasing the amount of PTFE nanofibrils the bubble walls become reinforced when expanded via a strain hardening effect, as described earlier in this paper. PTFE fibrils act against bubble wall fracture and give the necessary time for foam solidification by preventing a foam collapse. The best foam appears to be the foam with a minimal amount of halloysite (0.5%, for bubble nucleation) and a maximal amount of PTFE (3%, for bubble wall reinforcement and preventing a foam collapse).

The cell parameters (density, diameter) of foams are collected in Table 3. The foam of PLA-H(0.5)P(1) shows a significant decrease in cell size and increase in cell density, relative to the other foam samples. The cell density has remarkably risen from 1.13 × 10^5^ cells/cm^3^ for neat PLA foam to 9.41 × 10^5^ cells/cm^3^, accompanied by an approximate 22% reduction in average cell size (96.8 μm to 75.6 μm). A comparable number of cells were seen in FPLA-H(1)P(0.5) with a cell density of 7.65 × 10^5^ cells/cm^3^ and an average cell size of 83.75 μm. It was observed that when the PTFE and HNC content reached 3 wt%, the cell size developed and cell density dropped for FPLA-H(0.5)P(3) and FPLA-H(3)P(0.5), respectively. This phenomenon arises from the aggregation of nanofillers within the PLA matrix, as clearly depicted in the SEM image. For FPLA-H(3)P(0.5), the cell density reduced to 4.41 × 10^5^ cells/cm^3^ followed by an increase in average cell size of 107.1 μm. This is attributed to the presence of higher loading of HNC, which, therefore, leads to uncontrolled cell growth. Thus, the heterogeneous nucleation effect has a negative impact on the foaming of cells at higher filler loadings. Furthermore, an excessive concentration of fillers above optimal loading induces particle agglomeration, hence resulting in larger cell size, lower cell density, and non-uniform cell structure.

## 5. Conclusions

In this study, the effects of varying concentrations of HNC and PTFE on the PLA matrix on morphological, mechanical, thermal, and foaming properties were investigated. The surface morphology of neat PLA and composite materials with different proportions of HNC and PTFE showed varying fracture behavior. It was found that high PTFE concentrations led to aggregation, affecting the mechanical properties. However, HNC and PTFE fillers were effectively dispersed throughout the PLA matrix during melt compounding. The tensile studies showed that the addition of HNC and PTFE fillers increased tensile strength, tensile strain, and young’s modulus, compared to the neat PLA composite. However, higher concentrations of filler could lead to premature cracks and decreased structural rigidity. The addition of fillers also decreased elongation at break. The DSC results showed that the addition of fillers significantly influenced crystallization and melting temperatures of PLA. The crystallization time decreased with filler concentration, indicating a nucleating effect on PLA crystallization, potentially altering foaming behavior. Extensional flow tests showed strain hardening in PLA systems influenced by HNC and PTFE, with PTFE showing more effective behavior than HNC due to deformation and fibrillar entanglements. The cellular morphology of foamed PLA and PLA/HNC/PTFE nanocomposite foams showed improved cell morphology compared to neat PLA foam. The improvement of foaming while using in situ-generated nanofibrils of PTFE was expected by us. In our older research described in ref. [19,20,21,22,23] we demonstrated a strong strain hardening in extensional viscosity of nanocomposites consisting of nano-fibrillated PTFE, and we proved in ref. [23] that a strong dependence of foamability is connected to a significant strain hardening in extensional viscosity. There are two processes that are responsible for better foaming: first, the strain hardening prevents excessive bubble growth, and second, more time is gained for the solidification and stabilization of a foam. Here these effects are due to the inclusion of HNC and PTFE nanofibrils, which, in addition, enhance cell nucleation and proliferation. Consequently, the addition of HNC and PTFE improved the melt strength and viscosity of the polymer matrix, which led to reduced cell size and increased density. However, higher filler loadings resulted in uncontrolled cell growth and non-uniform cell structure.

## Figures and Tables

**Table 1:** Formulation of binary and ternary composite foams.

Samples	PLA (wt%)	HNC (wt%)	PTFE (wt%)	ADC (phr)
Neat PLA	100	-	-	3
PLA-H(0.5)	99	0.5	-	3
PLA-H(3)	97	3	-	3
PLA-P(0.5)	99	-	0.5	3
PLA-P(3)	97	-	3	3
PLA-H(0.5)P(0.5)	99	0.5	0.5	3
PLA-H(0.5)P(1)	98.5	0.5	1	3
PLA-H(0.5)P(3)	96.5	0.5	3	3
PLA-H(1)P(0.5)	98.5	1	0.5	3
PLA-H(3)P(0.5)	96.5	3	0.5	3

**Table 2:** Tensile properties of neat PLA and PLA/HNC/PTFE ternary composites with varying filler concentration. The accuracy was determined based on four tensile tests for each nanocomposite.

Sample	Tensile Strength (MPa)	Tensile Strain (%)	Young’s Modulus (MPa)
Neat PLA	49.3 ± 1.7	4.9 ± 0.7	1140 ± 43
PLA-H(0.5)P(0.5)	42.7 ± 1.3	3.4 ± 1.5	1005 ± 44
PLA-H(0.5)P(1)	59.1 ± 0.8	5.7 ± 0.9	1070 ± 50
PLA-H(0.5)P(3)	50.5 ± 0.5	4.8 ± 1.8	1275 ± 38
PLA-H(1)P(0.5)	53.3 ± 0.3	4.5 ± 0.6	930 ± 33
PLA-H(3)P(0.5)	49.2 ± 1.2	4.5 ± 1.7	1360 ± 43

**Table 3:** Representing the density of both composites and foams, cell density, and average cell diameter of ternary composite foams.

Sample	Density (g/cm^3^) (Composite)	Density (g/cm^3^) (Foam)	Cell Density (Cells/cm^3^)	Average Cell Diameter (µm)
Neat PLA	1.21	0.9	1.13 × 10^5^	96.89
PLA-H(0.5)P(0.5)	1.23	0.86	6.79 × 10^5^	83.06
PLA-H(1)P(0.5)	1.19	0.96	7.64 × 10^5^	83.75
PLA-H(3)P(0.5)	1.17	0.74	4.41 × 10^5^	107.1
PLA-H(0.5)P(1)	1.16	0.78	9.41 × 10^5^	75.63
PLA-H(0.5)P(3)	1.15	0.75	8.89 × 10^5^	78.46

## Data Availability

The original contributions presented in this study are included in the article, and further inquiries can be directed to the corresponding author.

## Abbreviations

The following abbreviations are used in this manuscript:

PLA	polylactide
HNC	halloysite nanoclay
PTFE	polytetraflouoroethylene
ADC	azodicarbonamide
SEM	scanning electron microscopy
